# Protecting the malaria drug arsenal: halting the rise and spread of amodiaquine resistance by monitoring the PfCRT SVMNT type

**DOI:** 10.1186/1475-2875-9-374

**Published:** 2010-12-23

**Authors:** Juliana M Sa, Olivia Twu

**Affiliations:** 1 Laboratory of Malaria and Vector Research, National Institute of Allergy and Infectious Diseases, National Institutes of Health, 12735 Twinbrook Parkway, Room 3E-10C, Rockville, MD, 20852, USA; 2 Dept of Microbiology, Immunology and Molecular Genetics, University of California Los Angeles, 1602 MSB, 609 Charles E. Young Dr. East, Los Angeles, CA, 90095, USA

## Abstract

The loss of chloroquine due to selection and spread of drug resistant *Plasmodium falciparum *parasites has greatly impacted malaria control, especially in highly endemic areas of Africa. Since chloroquine removal a decade ago, the guidelines to treat falciparum malaria suggest combination therapies, preferentially with an artemisinin derivative. One of the recommended partner drugs is amodiaquine, a pro-drug that relies on its active metabolite monodesethylamodiaquine, and is still effective in areas of Africa, but not in regions of South America. Genetic studies on *P. falciparum *parasites have shown that different *pfcrt *mutant haplotypes are linked to distinct levels of chloroquine and amodiaquine responses. The *pfcrt *haplotype SVMNT (termed after the amino acids from codon positions 72-76) is stably present in several areas where amodiaquine was introduced and widely used. Parasites with this haplotype are highly resistant to monodesethylamodiaquine and also resistant to chloroquine. The presence of this haplotype in Africa was found for the first time in 2004 in Tanzania and a role for amodiaquine in the selection of this haplotype was suggested. This commentary discusses the finding of a second site in Africa with high incidence of this haplotype. The >50% SVMNT haplotype prevalence in Angola represents a threat to the rise and spread of amodiaquine resistance. It is paramount to monitor *pfcrt *haplotypes in every country currently using amodiaquine and to re-evaluate current combination therapies in areas where SVMNT type parasites are prevalent.

## Background

The article "*Plasmodium falciparum *isolates from Angola show the StctVMNT haplotype in the *pfcrt *gene" by Gama BE *et al *[1] provides results that are more worrisome than surprising. This paper, along with a report from Tanzania [2] and a genetic study between two parasites from Africa and South America [3], highlights an urgent need to examine and update treatment guidelines that use combination therapies with 4-aminoquinolines.

Gama *et al *examined the prevalence of different haplotypes of the *Plasmodium falciparum *chloroquine (CQ) resistance transporter gene *pfcrt*, and the multiple drug resistance transporter *pfmdr1*, from patients in Angola with uncomplicated malaria in 2007. At that time the patients were treated with one of the two artemisinin combination therapies, artemether + lumefantrine or artesunate + amodiaquine (AQ), in accordance with the country's anti-malarial guidelines.

**Figure 1:**
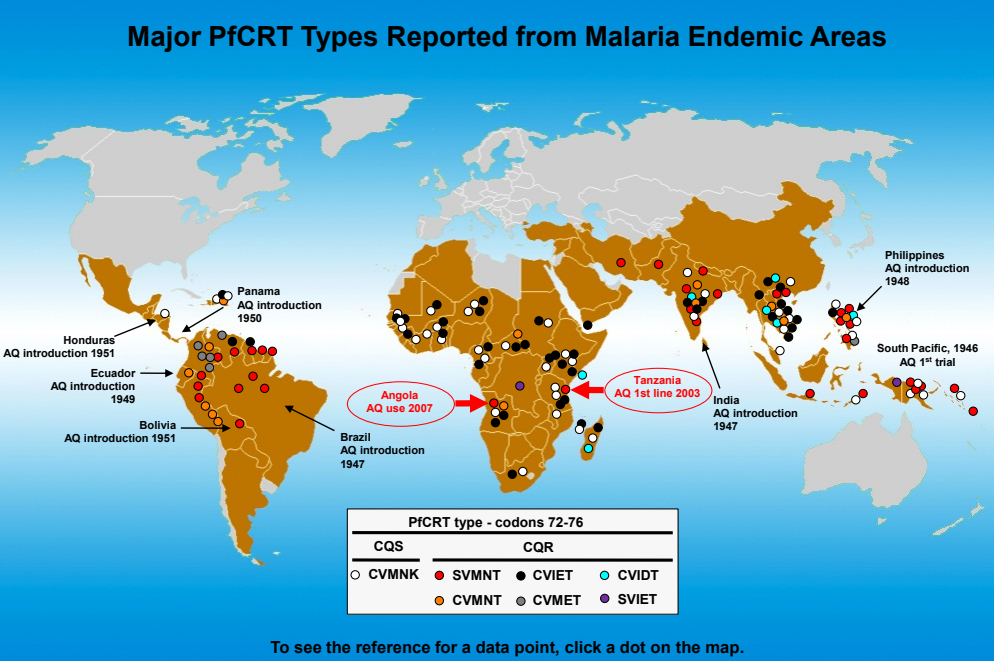
**Current worldwide distribution of PfCRT types (codons 72-76, based on the report of more than a single parasite isolate)**. Regions with previous history of AQ use coincide with areas where the SVMNT haplotype is prevalent. Figure adapted and updated from Sa *et al *[3].

The article reports on the "unexpected" prevalence (> 50%) of a CQ-resistant *pfcrt *haplotype often found in other countries including Brazil, India, Papua New Guinea, and the Philippines (termed SVMNT, based on codon positions 72-76, Figure 1). Since the characterization of *pfcrt *as the main genetic determinant of CQ resistance [4] and the removal of CQ from WHO anti-malarial guidelines in 2000, this is the second report of this haplotype in Africa. While Gama *et al *propose that the commercial relationship between Brazil and Angola allows for the import of the SVMNT haplotype through travellers, the report in 2006 by Alifrangis *et al *suggested a role of AQ in the selection of the SVMNT haplotype in Tanzania [2]. Interestingly, two other reports from the same countries show contrasting results regarding the *pfcrt *haplotype on those areas. The first, in Uige, Angola, shows that in 2004 no SVMNT type parasites were detected [5]. The second is a five-year follow up from Tanzania, in which no SVMNT haplotype was found in 2006 and 2007 after removal of AQ [6]. It is not clear whether differential patterns of parasite transmission levels in distinct geographic locations or drug policy changes may account for this "patchy" distribution of SVMNT type parasites. It is also important to note that after the spread of CQ resistance in Brazil and attempts to re-introduce AQ in the late 1980 s that country showed the highest level of AQ resistance reported at the time [7]. The appearance and spread of the SVMNT haplotype in Africa, where AQ has been widely used in the past decade in combination therapies should be more expected than surprising.

## Discussion

The analysis of the *in vitro *responses to CQ, AQ, and its active metabolite monodesethylamodiaquine (MDAQ) from the *P. falciparum *genetic cross 7G8xGB4, between a CQ-resistant clone from South America carrying the SVMNT *pfcrt *haplotype and an African clone carrying the CVIET haplotype, showed that these haplotypes are linked to distinct AQ/MDAQ and CQ responses [3]. Parasites with the SVMNT haplotype are highly resistant to MDAQ, but only moderately resistant to CQ, whereas clones with the CVIET haplotype are moderately resistant to MDAQ and highly resistant to CQ. These data, coupled with observations on the historical use of AQ with early trials in India, Brazil, the Philippines, Laos, Ecuador, Bolivia, Honduras, and areas of South Pacific (mostly matching the geographic distribution of the SVMNT haplotype, Figure 1), suggest that AQ had an early and prominent role in the selection of drug resistant SVMNT type parasites. Furthermore, data on the Tanzania genotypes of *P. falciparum *infected samples collected in 2003 and 2004, revealed SVMNT haplotype prevalence of 0% and 19%, after the increased use of AQ indicating a strong and quick evolutionary force. This idea is also supported by another recent report from Afghanistan, where the prevalence of *P. falciparum *SVMNT type has been associated with AQ resistance [8]. The mechanism by which different *pfcrt *haplotypes interact with AQ, MDAQ, and CQ is yet to be determined, but it may rely on their specific physicochemical characteristics such as amino acid side-chain volume, charge, and hydrophobicity [9].

A greater fitness cost may be associated with parasites carrying the CVIET rather than the SVMNT *pfcrt *haplotype. While regions in Africa and China where CVIET was the prevalent, if not exclusive haplotype, have been repopulated by CQ-sensitive parasites with the CVMNK haplotype after CQ removal [10-13] regions of South America have remained saturated by the SVMNT haplotype decades after CQ and AQ removal. A reduced fitness cost of the SVMNT haplotype suggests that once this haplotype is present, and CQ and AQ are removed, repopulation of sensitive strains may be very slow to occur.

It is essential to reiterate to the scientific community and government agencies the possibility of this hypothesis: AQ use results in the rapid evolution of 4-aminoquinoline resistant *P. falciparum *parasites of the *pfcrt *haplotype SVMNT, which easily adapt with relatively little fitness cost. The monitoring of *pfcrt *codon positions 72-76 from *P. falciparum *infected patients in all countries using AQ in their treatment guidelines, regardless of the combination drug is crucial. An estimation of the current distribution of parasites with this haplotype and the consideration of complete removal of AQ from areas of SVMNT prevalence is important because of the resistance levels of SVMNT parasites and the likelihood that they will be as fit as the drug-resistant parasites from South America, where the treatment options of AQ as well as CQ have long been lost.

## Conclusions

The continued use of AQ in combination therapies is dangerous in regions where resistant SVMNT parasites occur and threatens the selection of parasites resistant to the partner drug. All efforts should be made to monitor and to halt the selection of SVMNT parasites in Africa and develop new partners in effective combination therapies that will protect the anti-malarial arsenal.

## Abbreviations

*Pfcrt*: *P. falciparum *chloroquine resistance transporter gene; CQ: chloroquine; AQ: amodiaquine; MDAQ: monodesethylamodiaquine.

## Competing interests

The authors declare that they have no competing interests.

## Authors' contributions

JMS and OT wrote the manuscript. All authors have read and approved the final manuscript.
